# Clinical and ultrasonographic observations of functional and mechanical intestinal obstruction in buffaloes (*Bubalus bubalis*)

**DOI:** 10.14202/vetworld.2016.475-480

**Published:** 2016-05-14

**Authors:** Arafat Khalphallah, Nasr-Eldin M. Aref, Enas Elmeligy, Sayed F. El-Hawari

**Affiliations:** 1 Department of Animal Medicine, Faculty of Veterinary Medicine, Assiut University, Assiut, Egypt; 2 Veterinary Teaching Hospital, Faculty of Veterinary Medicine, Assiut University, Assiut, Egypt; 3 Department of Surgery, Anesthesiology and Radiology, Faculty of Veterinary Medicine, Sohag University, Sohag, Egypt

**Keywords:** buffalo, ileus, intestine, intussusception, ultrasonography

## Abstract

**Aim:**

This study was designed for clinical and laboratory evaluation of intestinal obstruction (IO) in buffaloes (*Bubalus bubalis*) with special emphasis on the diagnostic value of ultrasonographic findings.

**Materials and Methods:**

A total number of 30 buffaloes were included in the study and divided into 2 groups: Healthy (n=10) and diseased group (n=20). Diseased buffaloes were admitted to the Veterinary Teaching Hospital at Assiut University, Egypt, with a history of anorexia, abdominal pain, various degrees of abdominal distention, and absence or presence of scanty mucoid faces. These animals were subjected to clinical and ultrasonographic as well as laboratory examinations.

**Results:**

Based on ultrasonographic findings, various forms of IO were diagnosed. Functional obstruction, paralytic ileus, was diagnosed in 17 cases (85%) while mechanical IO was diagnosed only in 3 cases (15%). Out of 17 cases of paralytic ileus, both proximal and distal ileuses were successfully imaged in 8 and 9 cases, respectively. Proximal ileus was imaged from the right dorsal flank region as a single dilated loop of diameter >6 cm, while distal ileus was imaged as multiple dilated loops of diameter <6 cm. Mechanical obstruction due to duodenal intussusception was visualized as two concentric rings with outer echogenic wall and hypoechoic lumen. All cases of IO showed leukocytosis, hypoproteinemia, and increased activity of alkaline phosphatase and aspartate aminotransferase.

**Conclusion:**

Ultrasonography proved to be an essential tool for diagnosis and differential diagnosis of various forms of IO in buffaloes.

## Introduction

Intestinal obstruction (IO) represents an abdominal emergency that is potentially life-threatening to the affected animals. It is seen in all large animal species but is most common in horses [1]. Cattle are the most commonly affected ruminants and diagnosis in sheep and goats is rare (except for intestinal volvulus in lambs) [1]. According to our best knowledge, few available data about IO in buffaloes with no exact monitoring figure for its incidence or prevalence in Egypt.

Two common types of IO that interrupt the flow of ingesta have been recognized in large ruminants: Mechanical and functional [1,2]. Mechanical IO occurs due to a wide variety of causes and is characterized as being luminal or extraluminal. Luminal obstructions include hemorrhagic jejunitis (jejunal occlusion with blood clots); phytobezoars; cecocolic volvulus; impacted ingesta and atresia coli, recti, and ani. Extraluminal obstructions include intussusceptions, strangulation, and volvulus of the gastrointestinal tract as well as intestinal compression with an expanding abdominal mass such as lymphosarcoma or fat necrosis [3-5].

Unlike mechanical obstruction; functional obstruction, called ileus or paralytic ileus, occurs because of cessation of peristalsis movement of the intestinal tract. The inciting cause of functional obstruction is not well determined; however, they are often associated with dietary or management factors, phytobezoars, parasite infection, enteritis, peritonitis, or electrolyte abnormalities [1]. Paralytic ileus has no gross abnormality but is characterized by generalized intestinal hypomotility or atony. This condition occurs more frequently than mechanical obstruction and is common in pregnant and recently parturient cows [1]. Recently, this syndrome has been reported in camels [6] and buffalos [7]. Animals with paralytic ileus show unspecific clinical signs and rectal findings [2].

IO may compose a large proportion of abdominal emergency situations for the bovine specialists, and may occur under different management conditions that put challenges in making a definitive diagnosis. Although the case history, signalment, and physical examination are important to formulate a tentative diagnosis, other diagnostic tools such as radiography and ultrasonography should be included to reach an accurate diagnosis and an effective therapeutic plan in a timely and orderly manner.

Therefore, this study was designed to clinical and laboratory evaluate the syndrome of IO in buffaloes *(Bubalus bubalis)* with special emphasis on the diagnostic value of ultrasonographic findings.

## Materials and Methods

### Ethical approval

The present study was approved by the Institutional Animal Ethics Committee of The Faculty of Veterinary Medicine at Assiut University.

### Animals

The study was carried out on 30 buffaloes of different ages and sex. They were divided into two groups: Control group (n=10) and diseased group (n=20). The control group was selected from healthy non-pregnant female buffaloes belonged to a herd at the Veterinary Teaching Hospital (VTH), Faculty of Veterinary Medicine, Assiut University, Egypt. The diseased buffaloes were admitted to the VTH with a history of anorexia, decrease fecal output, and abdominal pain. Various degrees of abdominal distention, mucoid scanty faces, and reduction of milk production separately or collectively were also reported in some cases. All Institutional and National Guidelines for the care and use of animals were followed.

### Clinical examination

All buffaloes underwent a thorough clinical examination according to Cockcroft [8]. The general condition and demeanor, rectal temperature, heart rate, respiratory rate, and lung sounds were determined. Swinging and/or percussion auscultation on both sides of the abdomen and tests for a reticular foreign body and rectal palpation were also carried out. Animals were treated in accordance with guidelines established by the Faculty of Veterinary Medicine atAssiut University.

### Ultrasonographic examination

Ultrasonographic examination was performed according to Braun and Marmier [9]. Briefly, The small intestine (SI) of was examined ultrasonographically with a 3.5 MHz sector transducer (FF Sonic, Model UF-4000, Tokyo, Japan). Buffaloes were examined on the right side, from the tuber coxae to the eighth intercostal space (ICS) and from the transverse processes of the vertebrae to the linea alba. The appearance of loops of SI and their contents and motility were assessed.

### Blood sampling

Whole blood and serum samples were collected, and all precautions of sample collections and preparation for accurate evaluation of hematological and biochemical indices were taken into consideration according to Otter [10].

### Complete blood count assessment

A fully automated blood cell counter machine, Medonic CA620 Vet hematology analyzer–Sweden, was used to determine various hematological parameters. The differential leukocyte count was determined using four field meander method.

### Biochemical assays

A spectrophotometric method was adopted to determine serum concentrations of liver enzymes: Aspartate aminotransferase (AST), gamma-glutamyl transferase (γGT), alkaline phosphatase (ALP), and serum total protein. All kits and reagents were obtained from Spectrum Reagents (Egyptian Company for Biotechnology, Egypt).

### Statistical analysis

Data were analyzed using SPSS statistical software packaged program for windows version 10.0.1 (SPSS Inc., Chicago, IL, USA). All data were presented as mean±standard deviation. Analysis of variance of (one-way ANOVA) was performed and the significance level was set at p≤0.05.

## Results

### Clinical signs

The clinical signs varied according to nature of IO. Two conditions have been identified in this study (Table-1): Mechanical obstruction due to intussusceptions and paralytic ileus.

**Table-1:** Classification of intestinal obstruction in buffaloes based on ultrasonography findings.

Group	Number
I. Control	10
II. Disease	20
A. Mechanical obstruction	
Intussusception of duodenum	3
B. Paralytic ileus alone	
Distal ileus of the SI with partial obstruction	4
Distal ileus of the SI with complete obstruction	5
Proximal ileus with complete obstruction of SI	3
Proximal ileus with partial obstruction of SI	5

SI=Small intestine

All admitted cases showed some degree of depression, anorexia, and cessation of ruminal motility. Rectal examination revealed empty rectum and the presence of mucus and dilated loop(s) of the intestine. Buffaloes with intestinal intussusception showed signs of abdominal pain, lack of defecation, and lethargy but no abdominal distention. Rectal findings could not detect intussusception; however, distended loops of the SI were palpable. Slight systemic changes, including elevated heart rate (90±5 beats/min), respiratory rate (32±7/min), and congested mucous membranes, were observed. Buffaloes with paralytic ileus showed slight abdominal distension, absent peristalsis, and marked reduction of defecation. Ballottement with simultaneous auscultation and percussion of the right abdominal cavity revealed ping sound in four cases. Rectal palpation was unspecific; however, distended intestinal loop was palpable.

### Ultrasonographic findings

**Figure-1:**
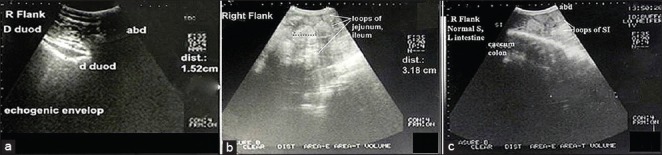
Ultrasonogram in 3-year-old healthy female buffalo imaged from right flank showed the descending part of duodenum of 1.5-4 cm Ø (echogenic wall and envelope) (a); loops of jejunum and ileum (two echogenic wall with echoic or hypoechoic contents and of 2.5-4.2 cm Ø) (b), and proximal loop of cecum and colon (c).

Ultrasonographic examination of the intestinal tract of the control group was conducted to setup a reference image on comparing with the diseased ones. Intestinal tract was imaged from the right flank region at different points of the last ICSs. The duodenum appeared as echogenic envelope with a diameter of 1.5-4 cm (Figure-1a) while the jejunum and ileum were imaged as loops with two echogenic wall in cross section with echoic or hypoechoic contents with a diameter of 2.5-4.2 cm (Figure-1b). The two walls (closest and furthest ones) of the SI were imaged as an echogenic wall. The cecum, proximal loop of the colon, and the spiral colon could be clearly imaged from the right flank region. The closest wall of the proximal loop of colon and cecum was imaged as a continuous or slightly curved echogenic line while the furthest wall of cecum and colon could not be imaged (Figure-1c).

**Figure-2:**
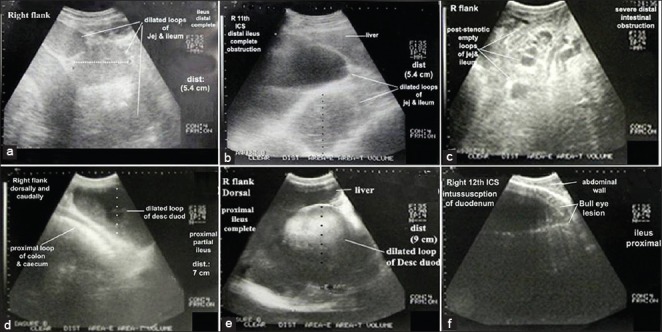
Ultrasonogram in a buffalo-heifer imaged from the right 11^th^ intercostal spaces (ICSs) and the right flank (RF) region suffered from distal ileus with complete obstruction. Small intestine showed multiple dilated loops of <6 cm Ø, two hyperechoic walls, echoic contents (a and b) and empty post-stenotic loops of ileum and jejunum with anechoic contents (c). Ultrasonogram in 5-year-old female buffalo imaged from the dorsal RF suffered from proximal ileus and partial obstruction (d) showed cross section (CS) in a dilated loop of the duodenum of >6 cm Ø (echogenic wall and envelope) with hypoechoic contents and anechoic lumen. Ultrasonogram in buffalo-bull suffered from proximal ileus with complete obstruction imaged from the ventral RF, middle 10^th^ and 11^th^ ICSs and caudodorsal RF showed CS in dilated loop of descending duodenum with cessation of the peristaltic movement (e). Ultrasonogram in 4-year-old buffalo-bull imaged from the RF showed CS dilated loop of the duodenal intussusception. Intussusception was visualized as two concentric rings with outer echogenic wall and hypoechoic lumen then inner highly reflective rings with anechoic center (f).

In diseased animals, two types of IO were identified based on ultrasound findings: Functional and mechanical. Cases of functional obstruction, ileus of the SI (n=17), could be distal at the area of the jejunum and ileum (distal ileus, n=9) or proximal at the area of the duodenum (proximal ileus, n=8) (Table-1). Ultrasonogram in distal ileus revealed the presence of multiple dilated loops of the jejunum and ileum with a diameter (Ø) of < 6 cm (4.5-6 cm), two hyperechoic walls and echoic contents (Figure-2a and b). The peristaltic movement was either reduced (partial obstruction, n=4) or cessated (complete obstruction, n=5) with empty post-stenotic loops of the ileum and jejunum with anechoic contents (Figure-2c).

On the other hand, ultrasonogram of proximal ileus showed the presence of one dilated loop of the duodenum with a diameter of >6 cm (Figure-2d and e). In proximal ileus with partial obstruction, the dilated loop of the descending part of the duodenum was clearly visualized as echogenic wall and envelop with hypoechoic contents and anechoic lumen (Figure-2d) along with the observation of normal loops of the jejunum and ileum as well as the proximal loop of the colon. The peristaltic movement was reduced. The dilated loop of the descending duodenum did not interfere with the liver or obscure the right kidney. In proximal ileus with complete obstruction (Figure-2e) dilated loop of the duodenum was imaged from the right with visualization of empty loops of the SI and complete reduction of the peristaltic movement of the SI.

Buffaloes with mechanical IO (n=3) due to intussusception were imaged from the right flank region in cross section as bull’s eyes lesion or bowel within bowel lesion (Figure-2f). The bull’s eyes lesion was visualized as two concentric rings with outer echogenic wall and hypoechoic lumen then inner highly reflective rings with the anechoic center.

### Blood picture and serum biochemical analysis (Table-2)

**Table-2:** Mean values±standard deviation of blood picture and serum biochemical indices in control and diseased buffaloes.

Parameter	Control	IO
T. WBCs (G/L)	6.71±1.63	17.35±3.6*
Neutrophils (%)	26.4±9.13	28.09±4.49
Lymphocytes (%)	60.80±7.73	48.42±6.69*
Monocytes (%)	7.80±4.63	19.04±2.6*
Eosinophils (%)	3.60±2.07	3.25±1.5
Band cells (%)	1.40±0.52	1.2±0.42
Total proteins (g/L)	94.7±10.7	68.9±5.9*
γGT (U/L)	14.95±5.23	18.14±3.03
ALP (U/L)	36.11±8.40	71.46±7.54*
AST (U/L)	32.92±4.77	84.34±8.92*

IO=Intestinal obstruction, T. WBC=Total white blood cell, γGT=Gamma-glutamyl transferase, ALP=Alkaline phosphatase, AST=Aspartate aminotransferase.

* Significant (p<0.05)

The hematological profiles in IO showed a significant increase (p<0.05) in the total leukocyte count. The population of individual WBC showed a significant decrease (p<0.05) in the percentage of lymphocytes and significant increase (p<0.05) in the percentage of monocytes. On the other hand, blood serum biochemical levels showed significant decrease p<0.05) in serum total protein and significant increase p<0.05) in blood serum activities of AST and ALP.

## Discussion

IO represents an abdominal emergency that is potentially life-threatening to the affected animals. IO could be tentatively diagnosed based on the results of the clinical examination. The first alarming sign associated with all admitted cases was no or significant reduction of fecal output, and the presence of mucus covering the figured fecal matter. Laboratory findings showed leukocytosis and hypoproteinemia, which could be attributed to prolonged anorexia, stress, and pain associated with IO [11]. The significant increase in the serum activities of AST and ALP could be attributed tissue destruction associated with intussusception [12]. These findings may have limited value in IO, and they do not reflect the exact diagnosis. Ultrasonography is an essential tool for diagnosis of several abdominal disorders [13]. In this study, ultrasonography was applied to diagnose IO and evaluate its forms in buffaloes. Differential diagnosis of various forms of IO in buffaloes based on ultrasonography findings was presented in Table-3. Imaging of the intestinal tract could be successfully performed from the right flank over the last 3 ICS. The descending part of the duodenum in healthy animal had a diameter of 1.7-4 cm, and the loops of jejunum and ileum had diameters of 2.5-4.2 cm. The normal peristaltic movement of the SI was clearly demonstrated. These findings agreed with that reported by Braun [13] in cattle. The closest wall of the proximal loop of the colon and cecum was also clearly imaged from right flank over the last 3 ICS as a continuous or slightly curved echogenic line while the furthest wall could not be imaged. Similar findings were reported by Braun and Amrein [14] in healthy cows.

**Table-3:** Differential diagnosis of various forms of intestinal obstruction in buffaloes based on ultrasonography findings.

Parameters	Intussusception	Proximal ileus obstruction		Distal ileus obstruction	
	
		Partial	Complete	Partial	Complete
Sites of the probe					
	Right abdominal side, from the tuber coxae to the 8^th^ ICS and from the transverse processes of the vertebrae to the linea alba				
Ultrasonogram					
Duodenum	Multiple concentric rings (Bull eye’s lesion), One loop >6 cm Ø	One dilated loop>6 cm Ø (9-15 cm)	One dilated loop> 6 cm Ø (9-15 cm)	Imaged normal	Nonvisualized
Jejunum and ileum	Imaged normal	Imaged normal	Nonvisualized	Multiple dilated loops<6 cm Ø (4.5-<6 cm)	Multiple dilated loops <6 cm Ø (4.5-<6 cm) Empty post-stenotic loops
Large intestine	Not imaged	Imaged normal	Not imaged	Imaged normal	Not imaged
Right kidney	Not imaged	Imaged normal	Not imaged	Imaged normal	Not imaged
Liver	Affection was intertangled with liver dorsally and occupied the ventral part of the last R-3 ICSs	Imaged Normal	Affection was intertangled with liver dorsally and occupied the ventral part of the last R-3 ICSs	Imaged normal	Affection was intertangled with liver dorsally and occupied the ventral part of the last R-3 ICSs
Peristaltic movement of SI	Reduced	Reduced	Ceased	Reduced	Ceased

ICSs=Intercostal spaces, SI=Small intestine

In comparison with healthy animals, ultrasound examination of IO due to ileus revealed different diameters and number of dilated loops of the duodenum, jejunum, and ileum according to the site of ileus. Proximal ileus showed the presence of one to five dilated loops of diameter >6 cm (9-15 cm) while distal ileus showed the presence of more than 5 dilated loops of diameter <6 cm (4.5-6 cm). These findings were in agreement with several studies conducted on cattle [15]. The authors reported that the larger diameter of dilated loops of SI; the more ileus of the proximal part of SI was indicated. In cows with proximal ileus, distal ileus at the jejunum and distal ileus at the ileum, the diameter of the intestine measured from the last right ICS varied from 6.5-9.9, 3.5-9.8, and 4.4-5.5 cm, respectively.

Mechanical obstruction due to intussusception of SI was also imaged just caudal to the last rib or last ICS at the level of the dorsal right flank region in a cross section as bull’s eye lesions. The bull’s eye lesions were visualized as two concentric rings with outer echogenic wall and hypoechoic lumen then inner highly reflective rings with the anechoic center. This ultrasonographic description agreed with that described in cattle [7,16]. The number of the dilated loops was only one loop at the site of intussusceptions suggests that the intussusception is in the duodenum.

Partial versus complete obstruction of the SI refers to the reduction or absence of peristaltic movement, respectively. In both proximal and distal partial obstruction, large intestine and right kidney could be visualized while they could not be imaged in the case of complete obstruction because dilated loops of the SI fully occupied the right flank region and the ventral part of the last right three ICS, and intertangled with liver lobes dorsally.

## Conclusion

It could be concluded that ultrasonography is a necessary tool for diagnosis of IO and to describe the nature and site of obstruction in buffaloes. The obtained results also suggested that functional obstruction (paralytic ileus) is a common form of IO in buffaloes.

## Authors’ Contributions

Authors have formulated the research plan. AK and NMA conducted clinical and ultrasonography examination, samples collection and analysis, and recorded the information. EE provided help in the analysis of data. SE helped in ultrasonography examination. AK drafted the manuscript and NMA revised it. All authors read and approved the final manuscript.
